# Is an Elevated Preoperative CRP Level a Predictive Factor for Wound Healing Disorders following Lumbar Spine Surgery?

**DOI:** 10.3390/jpm14070667

**Published:** 2024-06-21

**Authors:** Anatoli Pinchuk, Michael Luchtmann, Belal Neyazi, Claudia A. Dumitru, Klaus Peter Stein, Ibrahim Erol Sandalcioglu, Ali Rashidi

**Affiliations:** 1 Department of Neurosurgery, Otto-von-Guericke-University Magdeburg, 39120 Magdeburg, Germany; belal.neyazi@med.ovgu.de (B.N.); claudia.dumitru@med.ovgu.de (C.A.D.); klaus-peter.stein@med.ovgu.de (K.P.S.); erol.sandalcioglu@med.ovgu.de (I.E.S.); ali.rashidi@med.ovgu.de (A.R.); 2 Department of Neurosurgery, Heinrich-Braun-Klinikum, 08060 Zwickau, Germany; michael.luchtmann@hbk-zwickau.de

**Keywords:** postoperative wound infections, spinal surgery, CRP, influencing factors and risk factors

## Abstract

Postoperative wound infections are a prevalent concern among the hospital-associated infections in Europe, leading to prolonged hospital stays, increased morbidity and mortality, and substantial patient burdens. Addressing the root causes of this complication is crucial, especially given the rising number of spine surgeries due to aging populations. Methods: A retrospective analysis was conducted on a cohort of 3019 patients who underwent lumbar spine surgery over a decade in our department. The study aimed to assess the predictors of wound healing disorders, focusing on laboratory values, particularly inflammatory parameters. Results: Of the 3019 patients, 2.5% (N = 74) experienced deep or superficial wound healing disorders, showing the significant correlation between C-reactive protein (CRP) levels and these disorders (*p* = 0.004). A multivariate analysis identified several factors, including age, sex, hypertension, diabetes, cardiac comorbidity, surgical duration, dural injury, and blood loss, as being correlated with wound healing disorders. Conclusion: Demographic factors, pre-existing conditions, and perioperative variables play a role in the occurrence of adverse effects related to wound healing disorders. Elevated CRP levels serve as an indicator of increased infection risk, though they are not a definitive diagnostic tool for wound healing disorders.

## 1. Introduction

In the field of spinal surgery, there has been a notable increase in the frequency of surgical interventions, accompanied by a corresponding rise in postoperative complications. Factors such as patient age, surgical duration, and comorbidities have been identified as contributing elements to these complications [1,2]. Given the escalating rate of surgeries and the shifting age demographics of the populace, the evaluation of risk factors is assuming greater significance [1]. Postoperative wound infections rank as the third most prevalent type of nosocomial infection and present a challenge across various surgical disciplines [3]. Complications such as postoperative wound healing disorders or infections can result in exacerbated back pain, thereby compromising the overall success of the surgical intervention [4,5,6]. Infections subsequent to spinal surgery not only lead to heightened resource utilization but also cause additional financial burdens [5]. 

Prognostically significant risk factors for wound healing disorders [7,8] in spinal surgery [9] typically include diabetes mellitus, a prolonged surgical duration, and a body mass index (BMI) exceeding 35 kg/m^2^ [10,11]. Other factors such as the number of segments operated upon, the surgical approach and technique, the type of implants utilized, and surgeon expertise also play contributory roles [12]. Smoking, steroid usage, and perioperative transfusions are additionally recognized as critical risk factors [12].

Preoperative laboratory testing stands out as the foremost and simplest means of detecting inflammatory processes in the human body. Parameters such as leukocyte count, C-reactive protein (CRP) level, and erythrocyte sedimentation rate (ESR) are integral to this assessment [13,14,15,16,17]. 

In addition to demographic data, pre- and perioperative risk factors and patient-related variables were investigated in this study to assess their influence on wound healing disorders. Moreover, we examined the relationship between elevated CRP levels before surgery and the occurrence of postoperative wound healing disorders.

## 2. Materials and Methods

In this retrospective study, we examined a cohort of 3019 patients who underwent lumbar region surgery at the Department of Neurosurgery between 2008 and 2018. The spectrum of surgical indications ranged from degenerative spinal canal stenosis to acute traumatic vertebral body injuries.

During the patient assessments, we gathered detailed information on concurrent illnesses and prior lumbar spine surgeries. Preoperatively, depending on their clinical presentation, magnetic resonance imaging (MRI) was conducted to assess the spinal cord and nerves, while computed tomography (CT) was utilized to evaluate bony structures.

The exclusion criteria comprised patients with inflammatory joint and spine diseases, open fractures, and those undergoing further diagnostic or therapeutic interventions as part of follow-up examinations. Clinical data were extracted from a registry and corresponding patient files. Categorical variables such as sex, BMI, ASA classification, hypertension, diabetes mellitus, smoking status, cardiovascular diseases, chronic inflammation, meningeal injuries, the number of operated segments, the use of nonsteroidal anti-inflammatory drugs, glucocorticoid intake, the perioperative use of synthetic materials, and the specific surgical intervention were analyzed.

Additionally, we retrospectively analyzed preoperative laboratory parameters such as hemoglobin levels, erythrocyte sedimentation rates (ESRs), leukocyte counts, and C-reactive protein (CRP) levels, along with age, the number of affected segments, and intraoperative blood loss.

A postoperative wound infection was defined by the presence of one of the following criteria:

(a) Purulent discharge from the superficial wound area, possibly from below, or pus discharge from the internal drain.
(b) Patients experiencing postoperative pain and new complaints displaying typical signs of inflammation in the wound area, such as erythema and hyperthermia, along with serologically typical signs of infection, including a renewed increase in inflammatory parameters (CRP, leukocytes).
(c) In instances of the mentioned criteria, neurological complaints, or persistent lower back pain, MRI imaging was performed postoperatively to rule out potential complications related to wound healing or infection.

### Statistical Analysis

Quantitative variables were summarized using the mean and standard deviation (SD) or median [1st quartile, 3rd quartile] depending on the distribution model—whether normal or not. Categorical data were presented as absolute frequencies (n) and percentages (%). The analysis was intentionally conducted at a significance level of 0.05 to accommodate its exploratory nature. Thus, any obtained *p*-value below 0.05 was considered statistically significant within this framework. For unadjusted analyses, Fisher’s exact test was applied to the categorical variables and the robust *t*-test (Satterthwaite) was employed for the continuous variables, with log transformation applied when the variables exhibited a notable deviation from normal distribution. Spearman’s correlation coefficient was used for correlation analyses. In addition to employing Fisher’s exact test and the robust *t*-test, propensity score matching was conducted. The goal was to create control groups, extracting the patients with similar characteristics from the samples to analyze them for risk factors. Data storage and statistical analyses were performed utilizing the SAS University Edition software package 9.4 (SAS Institute, Inc., Cary, NC, USA).

## 3. Results

A total of 74 out of the 3019 patients were diagnosed with postoperative wound infections, resulting in an infection rate of 2.5%. Table 1 presents a comprehensive list of patients who experienced nosocomial wound infections, accompanied by an overview of the examined risk factors that may have led up to the occurrence of the infection. 

In our study, patients with wound healing disorders were an average age of 67.8 years old (*p* < 0.001), while those without postoperative wound complications were younger, with a mean age of 60.8 years. Gender emerged as a significant factor (*p* < 0.001). Among the 74 patients experiencing impaired wound healing, 77.03% (N = 57) were female and 22.97% were male (N = 17). However, other factors such as BMI and smoking status did not show a significant connection (BMI *p* = 0.101, smoker *p* = 0.173). Preoperative parameters, such as the ASA classification, demonstrated high significance (*p* < 0.001). Additionally, certain prior illnesses showed notable associations. Out of the 1697 patients with arterial hypertension, 57 patients experienced a postoperative wound infection (infection rate 3.35%) (*p* < 0.001). Among the 481 patients with diabetes mellitus, 22 patients (4.57%) developed a postoperative wound infection (*p* = 0.012). In the case of the 689 patients with coronary artery disease, 30 patients (4.35%) experienced a postoperative wound infection (*p* < 0.001).

The probability of a wound healing disorder in an individual with a healthy heart was significantly lower, at 1.88%. For chronic inflammations such as rheumatoid arthritis, there was no significant evidence (*p* = 0.078) of a connection. Differences were evident in the number of operated segments (*p* < 0.001). The majority of wound complications arose when two segments were operated on (54.79%, i.e., 40 patients). The likelihood of a wound complication increases with the number of operated segments: 1.14% with one segment, 3.16% with two segments, and 6.13% of patients with three or more segments operated on develop wound complications. Of the 535 patients with a dural injury, wound healing disorders subsequently occurred in 5.04% (N = 27) (*p* < 0.001). The risk of developing a wound complication was 2.1 times higher when using synthetic materials (*p* = 0.002). The intake of cortisone (*p* = 0.152) and non-steroidal anti-inflammatory drugs (*p* = 0.293) was not a significant indication of wound healing disorders. The incision–suture time ranged between 122.3 and 135.4 min (*p* < 0.001). Blood loss during surgery emerged as a significant predictor of wound healing (*p* < 0.002). The higher the blood loss, the more likely the risk of a wound healing disorder. The mean blood loss in patients with wound healing disorders was 217.9.

**Figure 1:**
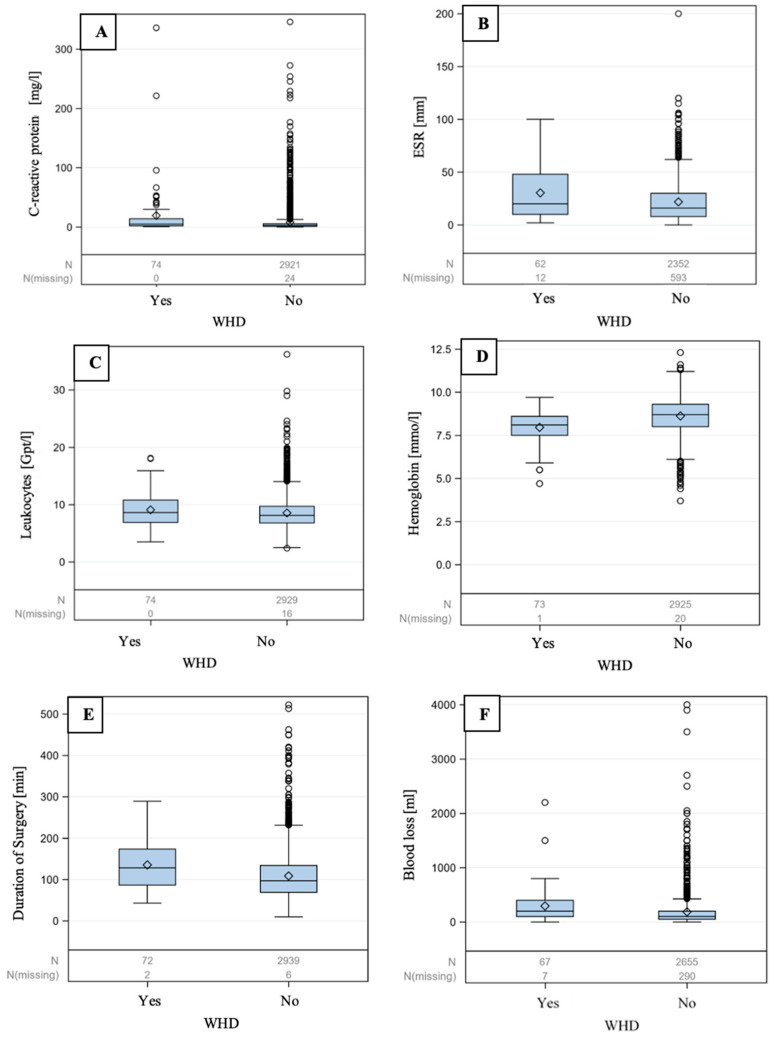
Boxplots corresponding to Table 3 depict continuous parameters in relation to wound healing disorders (WHDs). Laboratory parameters and operative characteristics that influence wound healing after surgery are C-reactive protein (**A**), erythrocyte sedimentation rate (**B**), leukocytes (**C**), hemoglobin (**D**), duration of surgery (**E**) and blood loss (**F**).

All patients with elevated CRP levels are summarized in Table 2 according to their previous illnesses. It becomes evident that many patients had an elevated CRP level without previous illnesses. Laboratory parameters, such as the CRP level (Table 3, Figure 1A), indicate clear evidence of a connection with wound healing disorders (*p* = 0.004). Patients with wound healing disorders had an average preoperative CRP value of 10.4 mg/L, whereas the mean for patients without wound complications was 4.8 mg/L. Blood sedimentation (Table 3, Figure 1B) also exhibited a significant correlation (*p* = 0.008). The mean value of the ESR in patients without wound healing disorders was 18.3 mm, whereas it increased to 25.5 mm in patients with wound healing disorders. The laboratory parameter of the small blood count, specifically the leukocyte count, showed no statistical significance (*p* = 0.117) (Table 3, Figure 1C). However, the mean amount of hemoglobin (Table 3, Figure 1D) in patients with wound healing disorders is 0.7 mmol/L lower (7.9 mmol/L) compared to patients without wound healing disorders (*p* = 0.001).

In patients with a postoperative wound infection, the average duration of the operation was 128.9 min (Table 3, Figure 1E). This is 26.3 min longer (102.6 min) than in patients who demonstrated good wound healing postoperatively. The incision–suture time ranged between 122.3 and 135.4 min (*p* < 0.001). Blood loss during surgery emerges as a significant predictor of wound healing (*p* < 0.002). The higher the blood loss, the more likely the risk of a wound healing disorder. The mean blood loss in patients with wound healing disorders was 217.9. In contrast, patients without wound complications exhibited significantly less blood loss, with an average of 125.1 mL (Table 3, Figure 1F). 

**Figure 2:**
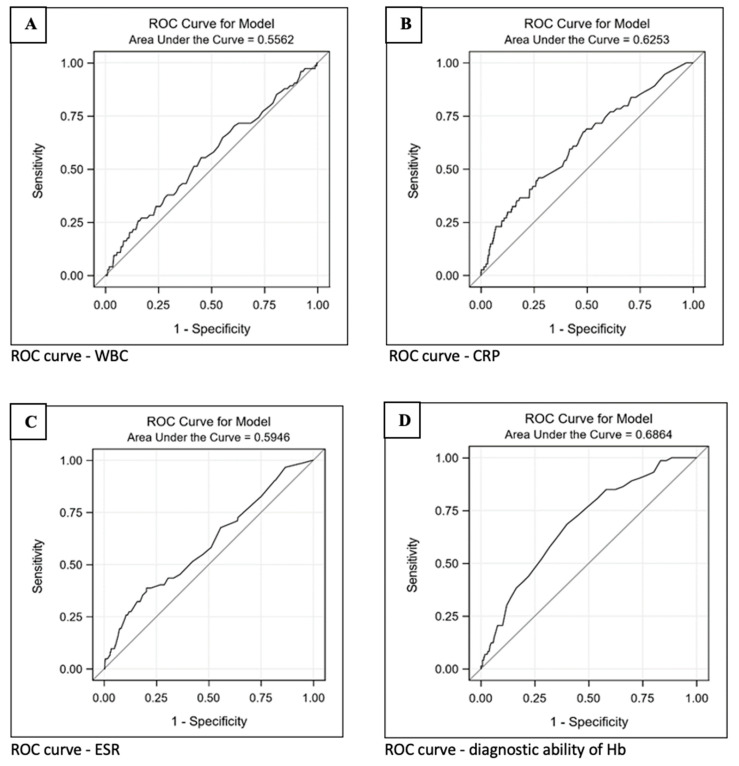
ROC analyses demonstrate sensitivity and specificity for distinguishing patients with and without wound healing disorders/infections using key laboratory parameters. The biomarkers leukocytes (**A**), C-reactive protein (**B**) and erythrocyte sedimentation rate (**C**) are considered as unsuitable diagnostic tools in this context. The best AUC is achieved for hemoglobin (**D**), although it does not have a particularly high diagnostic quality.

For each conceivable cutoff of the variables, receiver operating characteristic (ROC) analyses compare their sensitivity and specificity in distinguishing between patients with and without wound healing disorders/infections. The criteria for differentiation stipulate that a higher value (above the cutoff) indicates wound healing disorders or infections. This assumption is only partially applicable to leukocytes (Figure 2A), as low values (<3.7) can also be pathological. However, these instances are infrequent compared to the values defined as pathological, which are >9.9, and have been disregarded. The area under the curve (AUC) for the CRP level (Figure 2B) is 0.625 (0.559; 0.692). The AUC for the ESR (Figure 2C) is 0.595 (0.520; 0.669). Both biomarkers are deemed unsuitable as diagnostic tools in this context. The best AUC is achieved for Hb (Figure 2D), although at 0.686 [0.629; 0.744], it does not exhibit a particularly high diagnostic quality.

## 4. Discussion

In this study, we conducted a retrospective analysis to identify and explore the factors influencing postoperative infections subsequent to spinal neurosurgical interventions over a decade. Comparatively, the incidence of infections following spinal neurosurgical procedures appears to be less frequent than those following cranial procedures in extant studies [18]. 

However, the reported infection rates in the literature exhibit wide variability, ranging from 0.9% to 5%, with a mean infection rate of 2.48% [19,20,21,22,23,24,25,26]. This variability can be attributed to differences in the patient populations across studies, as well as variations in surgeon experience, operative areas, and surgical techniques. The infection rate of wound infections in our study was 2.5%, aligning with the mean of corresponding studies.

Patients with wound healing disorders showed a slightly elevated CRP level, with an average value of 10.4 mg/L. In contrast, the CRP level in patients without wound complications was 4.8 mg/L. Elevated levels of the erythrocyte sedimentation rate, hemoglobin, and CRP have been indicative of an increased risk of infection [27,28,29]. The preoperative CRP value proves to be valuable in assessing the risk of postoperative infections [30,31,32,33,34]. This was also confirmed in our study. In another study, no significant influence of the CRP level on infection was observed [35]. It is pertinent to note that elevated CRP values may be present in various pre-existing diseases and conditions, as well as post-operative procedures unrelated to the manifested infections [16,36]. The ESR demonstrated a significant association with wound healing disorders and was approximately 25.5 mm higher in patients with complications compared to those without (18.3 mm). There is a significant body of literature critically assessing the ESR value for postoperative wound infection assessment [37,38]. Some publications suggest a specific postoperative pattern of ESRs, reporting a regular postoperative increase similar to CRP values and recommending critical evaluation if the value does not decrease after one week [39]. In the current study, ESR and CRP values were analyzed preoperatively as a benchmark for infection risk, and we observed that elevated levels of both factors are associated with increased infection risk. Despite the significant association of elevated CRP and ESR values with the development of wound healing disorders, the ROC analysis, which compares their sensitivity and specificity in distinguishing between patients with and without such disorders or infections, suggested that these two biomarkers may not be deemed suitable diagnostic aids for wound healing disorders.

While some studies emphasize the importance of inflammatory parameters such as leukocytes for the early detection of postoperative complications after lumbar disc surgery [40], our results did not show a statistically significant connection between leukocytes and wound healing disorders (*p* = 0.117). The leukocyte count in patients with wound complications was 8.9 gpt/L, compared to 8.4 gpt/L in those without complications. Our findings are in line with Jenny et al., who argued that leukocyte determination is not a meaningful indicator for the diagnosis of infection [38,41]. 

Obesity is widely recognized by most authors as a risk factor for postoperative infections after spinal surgery [10,42]. The data from our study align with this perspective: while only 1.76% of normal-weight patients experienced a postoperative wound infection, the rate increased to 3.52% for patients with grade I–III obesity. In the literature, the male gender is often associated with a higher risk of postoperative infections and is considered a predictive risk factor [43]. However, conflicting studies argue that sex may not have a significant influence on postoperative infections’ development [44,45]. In contrast to these findings, our study revealed that 1.08% of male patients developed a postoperative wound infection, whereas the rate was higher, at 3.93%, for female patients. In terms of percentages, the females experienced postoperative wound infections more frequently. Several studies [46,47] have demonstrated a positive correlation between age and the occurrence of postoperative wound infections after spinal surgery. This correlation is affirmed in our study, where the patients without wound complications were an average age of 60.8, while those with wound healing disorders were significantly older (67.8). The presence of previous illnesses or comorbidities has been linked to an increased rate of postoperative complications by various authors, and our data confirm this correlation [48,49]. The ASA score has been associated with postoperative wound infections in some studies [50,51]. In our study, the probability of postoperative wound healing disorders was 5.37% with an assigned ASA score of 3–4. Generally, a higher ASA score corresponds to a greater likelihood of a wound healing disorder.

Further studies [6,47,52] have shown that diabetes mellitus is a risk factor for postoperative wound infections. Among the 481 patients with insulin-dependent and insulin-independent diabetes mellitus in our study, 22 patients (4.57%) experienced a postoperative wound infection. The diabetic patients were more than twice as likely to develop a wound healing disorder (2.06% in non-diabetics vs. 4.57% in diabetics). In a study by Haleem et al. [53] involving 2309 patients (2.3% infection rate), after the decompression of the spine, arterial hypertension was shown to be a significant risk factor for the occurrence of postoperative wound infection, consistent with our study. Out of the 1640 patients with a prior diagnosis of arterial hypertension, 57 patients (an infection rate of 3.35%) experienced a nosocomial wound infection. A robust association between prior cardiac conditions and the occurrence of complications or difficulties in wound healing has been demonstrated [54,55]. Cardiovascular diseases negatively impact the wound healing process due to altered metabolic conditions and reduced tissue perfusion. In our study, patients with heart disease were more than twice as likely to develop a wound healing disorder compared to those without heart disease (4.35% for heart disease patients vs. 1.88% for heart-healthy patients). A crucial factor in the development of deep wound infections is an injury to the dura mater. This is a common complication of lumbar surgery, with an incidence ranging from 0.2 percent to 20 percent [56]. In our presented study, the complication rate of dural opening was 21.5 percent (535 patients). The risk of developing a wound healing disorder after dural opening is almost three times higher (5.04% with dural opening vs. 1.89% without dural opening). One possible explanation is the delayed mobilization of patients, leading to an increased risk of immobility-related complications or an additional stimulus for proliferative processes in the epidural space due to cerebrospinal fluid leakage.

As an additional intraoperative measure, synthetic materials were used in 22.95% of patients undergoing duraplasty as a necessary therapy for intraoperative durotomy. This prolonged the operation time and simultaneously led to a significantly increased risk of postoperative complications. The risk of developing a wound complication is more than two times higher when using exogenous materials (4.11% with synthetic materials vs. 1.94% without synthetic materials). The use of fibrin glue is discussed in the literature, but there is no evidence of an increased infection rate when using fibrin glue [47].

Our study revealed a significant correlation between the number of operated segments and the occurrence of postoperative wound infections (1.14% with one operated segment vs. 6.13% with at least three operated segments). In the literature, this criterion is described as a known risk factor for the development of wound complications after spinal surgery [7]. This is justified by the extended duration of the operation and the increased volume of damaged soft tissue, which could lead to postoperative complications.

A longer duration of the operation significantly increases the risk of infection [6,57], primarily due to increased bacterial wound colonization [58]. The duration of the operation depends on the intervention, the complexity, and the skill of the surgeon. In this study, the average duration of surgery in patients with wound healing disorders was 128.9 min, while patients without wound complications had a shorter operation time (102.6 min).

Our work demonstrated that patients with postoperative infections lose almost twice as much blood as patients without wound healing disorders (217.9 mL vs. 125.1 mL). As mentioned earlier, the literature links the duration of the operation to the volume of blood loss: the longer the operation, the higher the blood loss [7,47]. The risk of impaired wound healing is attributed to the reduction in hemoglobin levels, resulting in decreased tissue oxygenation. 

## 5. Conclusions

Elevated inflammatory parameters, particularly preoperative CRP levels, were indicative of postoperative wound healing disorders or infections. However, it is important to note that these laboratory parameters cannot be considered predictive factors for wound healing complications. The determinants contributing to an elevated CRP level should be elucidated through a separate study. Identifying significant risk factors allows for targeted interventions aimed at infection prophylaxis in high-risk cases. Since the conclusion of our study, patients with elevated inflammatory parameters have had their elective surgery postponed until a thorough focus search is conducted, and, if necessary, preoperative antibiotic treatment is initiated.

In our view, a prospective, short-term, multicenter study should be conducted to gather data from a large cohort of cases and provide a more precise assessment. Subsequent data analyses from these various centers could aid in the development of guidelines.

## Figures and Tables

**Table 1:** Among the demographic data and comorbidities, sex, age, ASA classification, hypertension, diabetes, and cardiovascular diseases have a significant influence on postoperative wound healing disorders. Among the operative characteristics, the following have a significant influence on postoperative wound healing disorders: (injury of the dura during surgery; number of operating segments; use of synthetic materials; duration of the operation [minutes]; blood loss during surgery [mL]).

Parameters		Wound Healing Disorder/Infection	No Wound Healing Disorder/Infection	*p*-Value
		N (%)	N (%)	
		Mean ± SD	Mean ± SD	
**Demographic data**				
Sex	Female	57 (77.03)	1392 (47.27)	<0.001
	Male	17 (22.97)	1553 (52.73)	
Age		74 (67.8 ± 11.7)	2929 (60.8 ± 14.8)	<0.001
ASA Classification	I	1 (1.39)	361 (12.30)	<0.001
	II	31 (43.06)	1869 (63.70)	
	III–IV	40 (55.56)	704 (23.99)	
Smoker	Yes	13 (17.57)	741 (25.16)	0.173
	No	61 (82.43)	2204 (74.84)	
BMI	Normal weight	12 (16.22)	679 (23.08)	0.101
	Pre-obesity	25 (33.78)	1185 (40.28)	
	Obesity grade I–III	37 (50.00)	1049 (35.66)	
**Comorbidities**				
Diabetes	Typ I	0 (0.00)	9 (0.31)	0.012
	Typ II	22 (29.73)	472 (16.03)	
	No	52 (70.27)	2464 (83.67)	
Hypertension	Yes	57 (77.03)	1640 (55.69)	<0.001
	No	17 (22.97)	1305 (44.31)	
Cardiovascular diseases	Yes	30 (40.54)	659 (22.38)	<0.001
	No	44 (59.46)	2286 (77.62)	
Chronic inflammation	Yes	8 (10.81)	170 (5.77)	0.078
	No	66 (89.19)	2775 (94.23)	
**Operative Characteristics**				
Injury of the dura during surgery	Yes	27 (36.49)	508 (17.25)	<0.001
	No	47 (63.51)	2437 (82.75)	
Number of operating segments	1	17 (23.29)	1466 (49.93)	<0.001
	2	40 (54.79)	1225 (41.72)	
	≥3	16 (21.92)	245 (8.34)	
Use of synthetic materials	Yes	29 (39.19)	676 (22.95)	0.002
	No	45 (60.81)	2269 (77.05)	
Duration of the operation [minutes]		72 (128.9)	2655 (125.1)	<0.001
		[122.3; 135.4]	[63.1; 187.0]	
Blood loss during surgery [mL]		67 (217.9)	2655 (125.1)	0.002
		[140.1; 295.6]	[63.1; 187.0]	

**Table 2:** Previous illnesses with elevated CRP values (>10.5 mg/L), including overlapping conditions. Among patients with arterial hypertension, 34.36% also had diabetes mellitus, while 12.27% were additionally affected by chronic inflammation.

Illnesses	N	%
Arterial hypertension	326	66.8
Chronic inflammation	145	29.7
Diabetes mellitus	123	25
Dyslipoproteinemia	4	0.82
Patients without previous illnesses	137	28

**Table 3:** Laboratory parameters and operative characteristics that influence wound healing after surgery. Giga particle (Gpt).

	Wound Healing Disorder/Infection		
	N/Mean [SD]		
	Yes	No	*p*-Value
Duration of the surgery [minutes]	72/128.9 [122.3; 135.4]	2939/102.6 [96.5; 108.7]	<0.001
Blood loss [mL]	67/217.9 [140.1; 295.6]	2655/125.1 [63.1; 187.0]	0.002
Blood sedimentation rate [mm]	62/25.5 [20.6; 30.5]	2352/18.3 [14.6; 21.9]	0.008
Hemoglobin [mmol/L]	73/7.9 [7.9; 8.0]	2925/8.6 [8.6; 8.6]	<0.001
C-reactive protein [mg/L]	74/10.4 [1.2; 19.6]	2921/4.8 [1.3; 8.2]	0.004
Leukocytes [gpt/L]	74/8.9 [8.6; 9.1]	2929/8.4 [8.2; 8.5]	0.117

## Data Availability

The datasets obtained and analyzed during the current study are available from the corresponding author on reasonable request.
